# Performance of the Applied Biosystems HIV-1 Genotyping Kit with Integrase

**DOI:** 10.1128/jcm.00136-24

**Published:** 2024-05-10

**Authors:** Hannah P. Moore, Philip J. Palumbo, Kin Israel Notarte, Jessica M. Fogel, Vanessa Cummings, Theresa Gamble, Carlos Del Rio, D. Scott Batey, Kenneth H. Mayer, Jason E. Farley, Robert H. Remien, Chris Beyrer, Sarah E. Hudelson, Susan H. Eshleman, for the HPTN 078 study

**Affiliations:** 1 Department of Pathology, Johns Hopkins University School of Medicine, Baltimore, Maryland, USA; 2 FHI 360, Durham, North Carolina, USA; 3 Department of Medicine, Emory University School of Medicine, Atlanta, Georgia, USA; 4 School of Social Work, Tulane Universtiy, New Orleans, Louisiana, USA; 5 Department of Medicine, Harvard Medical School, Boston, Massachusetts, USA; 6 Fenway Institute, Boston, Massachusetts, USA; 7 The Center for Infectious Disease and Nursing Innovation, Johns Hopkins University School of Nursing, Baltimore, Maryland, USA; 8 HIV Center for Clinical and Behavioral Studies, New York State Psychiatric Institute, New York, New York, USA; 9 Department of Psychiatry, Columbia University, New York, New York, USA; 10 Global Health Institute, Duke University, Durham, North Carolina, USA; The University of North Carolina at Chapel Hill School of Medicine, Chapel Hill, North Carolina, USA

**Keywords:** HIV, genotyping, Applied Biosystems, drug resistance, mutations, next-generation sequencing

## Abstract

HIV genotyping is used to assess HIV susceptibility to antiretroviral drugs. The Applied Biosystems HIV-1 Genotyping Kit with Integrase (AB kit, Thermo Fisher Scientific) detects resistance-associated mutations (RAMs) in HIV protease (PR), reverse transcriptase (RT), and integrase (IN). We compared results from the AB kit with results obtained previously with the ViroSeq HIV-1 Genotyping System. DNA amplicons from the AB kit were also analyzed using next-generation sequencing (NGS). HIV RNA was extracted using the MagNA Pure 24 instrument (Roche Diagnostics; 96 plasma samples, HIV subtype B, viral load range: 530–737,741 copies/mL). FASTA files were generated from AB kit data using Exatype (Hyrax Biosciences). DNA amplicons from the AB kit were also analyzed by NGS using the Nextera XT kit (Illumina). Drug resistance was predicted using the Stanford HIV Drug Resistance Database. The mean genetic distance for sequences from ViroSeq and the AB kit was 0.02% for PR/RT and 0.04% for IN; 103 major RAMs were detected by both methods. Four additional major RAMs were detected by the AB kit only. These four major RAMs were also detected by NGS (detected in 18.1%–38.2% of NGS reads). NGS detected 27 major RAMs that were not detected with either of the Sanger sequencing-based kits. All major RAMs detected with ViroSeq were detected with the AB kit; additional RAMs were detected with the AB kit only. DNA amplicons from the AB kit can be used for NGS for more sensitive detection of RAMs.

## INTRODUCTION

Antiretroviral therapy (ART) is highly effective for reducing HIV transmission and HIV-associated morbidity and mortality among people living with HIV (1, 2). HIV drug resistance is the primary cause of treatment failure (3, 4) and can decrease the effectiveness of antiretroviral (ARV)-based regimens for HIV pre-exposure prophylaxis (PrEP). Improvement in the global availability of ARV drugs for HIV treatment and prevention has led to increased HIV drug resistance, especially to regimens based on non-nucleoside reverse transcriptase inhibitors (NNRTIs) (5–7). HIV drug resistance testing in clinical settings is needed to inform optimal ART selection. Surveillance of drug resistance is also important for monitoring the prevalence and type of drug resistance in different settings, which can inform HIV treatment and prevention programs.

HIV genotyping can be performed using commercial kits or laboratory-developed tests. Some methods use Sanger sequencing to identify resistance-associated mutations (RAMs); next-generation sequencing (NGS) can also be used for HIV genotyping and is more sensitive for detection of RAMs. In 2021, the Sanger-based, U.S. Food and Drug Administration (FDA)-cleared ViroSeq HIV-1 Genotyping System (ViroSeq; Abbott Molecular, Abbott Park, IL) was discontinued by the manufacturer. In 2022, Thermo Fisher Scientific (Waltham, MA) introduced the Applied Biosystems HIV-1 Genotyping Kit with Integrase (AB kit), which similarly uses Sanger sequencing. This product is labeled for Research Use Only and is available globally. A second kit offered by Thermo Fisher Scientific, the Applied Biosystems TaqPath Seq HIV-1 Genotyping Kit, carries the Conformité Européenne *In Vitro* Diagnostic Directive (CE IVDD) mark, is labeled for *in vitro* diagnostic use, and is available in countries that recognize this designation. The formulation and manufacturing process of these two kits are identical. These two kits would be expected to have the same performance characteristics. In this report, we evaluated the performance of the AB kit using samples from a clinical trial in the United States that were previously analyzed with ViroSeq (8). DNA amplicons generated with the AB kit were also analyzed using a laboratory-developed NGS method.

## MATERIALS AND METHODS

### Samples used for analysis

The HIV Prevention Trials Network (HPTN) 078 study (NCT02663219) enrolled men who have sex with men and transgender women from four US cities (Atlanta, GA; Baltimore, MD; Birmingham, AL; Boston, MA) (9). The study evaluated an intervention aimed at improving linkage to care, ART initiation, treatment adherence, and retention in care. Most (86.1%) of the participants in HPTN 078 reported having taken ART at study entry (9). HIV drug resistance was detected at study entry in 31.0% of the participants with genotyping results; detection of drug resistance was associated with self-reported prior ART in a multivariate model (8). This sample set was selected for analysis because it included a high prevalence of RAMs, including integrase strand transfer inhibitor (INSTI) RAMs. The study included all participants who had a sufficient volume of stored plasma available for analysis (from study screening or enrollment; one sample per participant). Plasma samples were stored at −80°C for up to 7 years before testing with the AB kit. Testing was performed using plasma aliquots that had not been previously thawed. These samples were analyzed previously using the RealTime HIV-1 Viral Load Assay (Abbott Molecular) and ViroSeq (8).

### Overview of laboratory testing for this study

**FIG 1:**
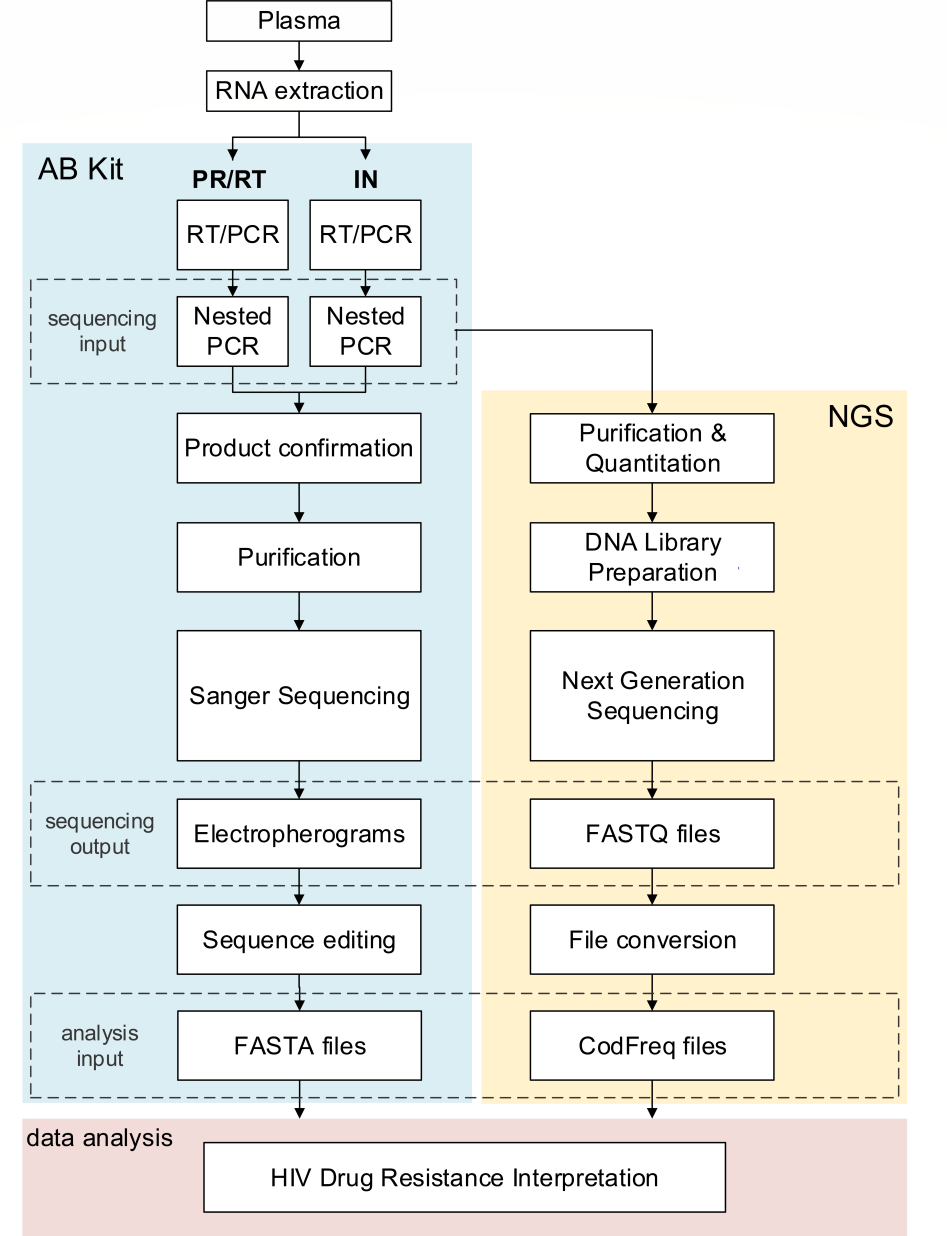
Methods used for HIV drug resistance analysis. The figure shows the workflow for HIV genotyping for the AB Kit (Thermo Fisher Scientific) and for NGS (see Methods). For both methods, RNA is first extracted from plasma. The regions of HIV PR/RT and IN are then reverse transcribed and amplified in separate reactions using primers from the AB Kit. Primers from the AB kit are used for a nested amplification reaction. The resulting DNA amplicons are verified by gel electrophoresis before sequencing. Analysis with the AB Kit (blue shading) includes amplicon purification and Sanger sequencing reactions using six primers for PR/RT and four primers for IN. Cycle sequencing products are purified and analyzed by capillary electrophoresis. Electropherograms are edited using the online Exatype platform (Hyrax Biosciences) to produce the final FASTA file for each sample. Analysis with NGS (yellow shading) is performed using the Nextera XT DNA Library Preparation Kit (Illumina). This analysis includes DNA purification, DNA quantification, library preparation, and sequencing. FASTQ sequence files are converted to CodFreq files. FASTA files from the AB Kit and CodFreq files from NGS are used for the interpretation of drug resistance (red shading) using the Stanford HIV Drug Resistance Database.

Laboratory testing was performed at the HPTN Laboratory Center (Johns Hopkins University, Baltimore, MD). Figure 1 describes the workflow for the analysis of samples using the AB kit and a laboratory-developed NGS method.

### HIV genotyping with the AB kit

HIV genotyping of protease (PR), reverse transcriptase (RT), and integrase (IN) was performed using the AB kit. This kit provides sequence information for PR codons 6–99, RT codons 1–251, and IN codons 1–288. Briefly, RNA was extracted from 500 µL of plasma using the MagNA Pure 24 System (Roche Diagnostics, Indianapolis, IN). The extraction yielded 50 µL of RNA. Using primers for PR/RT or IN, 10 µL of extracted RNA was reverse transcribed and amplified by polymerase chain reaction (PCR); nested PCR was performed for each region using two µL of amplified DNA. DNA amplicons were verified by gel electrophoresis and purified using ExoSAP-IT (Thermo Fisher Scientific). Sanger sequencing reactions were performed using six primers for PR/RT and four primers for IN; 2 µL of purified DNA was used for each reaction. Cycle sequencing products were purified using the BigDye XTerminator Purification Kit (Thermo Fisher Scientific) and analyzed by capillary electrophoresis using the Applied Biosystems 3500xL Genetic Analyzer (Thermo Fisher Scientific). Electropherograms were uploaded to a sequence-editing platform (Exatype, Hyrax Biosciences, Capetown, South Africa). Software-guided editing and manual editing were performed and consensus sequences (FASTA files) were generated. Samples that failed Sanger sequencing for one or both regions were initially re-sequenced using purified nested PCR products; if this was not successful, samples were reanalyzed using extracted RNA.

Analysis of HIV drug resistance for nucleoside/nucleotide reverse transcriptase inhibitors (NRTIs), NNRTIs, protease inhibitors (PIs), and INSTIs was performed using the Stanford HIV Drug Resistance Database (v9.5.0). For this analysis, FASTA files from the AB kit and ViroSeq were uploaded to “Input sequences” with the output options of “sequence summary” and “resistance summary”. Data were categorized by HIV subtype, RAMs detected, and predicted HIV drug resistance.

### Next-generation sequencing

Amplicons generated with the AB kit were also analyzed using a laboratory-developed NGS method. Briefly, 10 µL of unpurified DNA amplicons generated with the AB kit was purified using AMPure XP beads (Beckman Coulter, Pasadena, CA). The purified DNA was quantified using a Qubit Fluorometer (Thermo Fisher Scientific). Purified PR/RT and IN amplicons were pooled in equal concentrations. Library preparation was performed using the Nextera XT DNA Library Preparation Kit (Illumina, San Diego, CA). Barcoded DNA libraries were pooled (24 samples maximum per run) and sequenced using the MiSeq System (2 × 300 cycles; reagent kit version 3; Illumina). Analysis of HIV drug resistance was performed using the “HIVdb Program: Sequence Reads (NGS) Analysis” program accessed on the Stanford HIV Drug Resistance Database (https://hivdb.stanford.edu/hivdb/by-reads/). For this analysis, FASTQ files with paired-end reads were converted to codon frequency files using the “Input sequence reads” and “Convert FASTQ → CodFreq Files” tools. Codon frequency files were analyzed using the “Input sequence reads” tool with the following output options: machine-readable data; minimum read depth, ≥50; nucleotide mixture threshold, “don’t apply”; and mutation detection threshold, ≥2%. Resulting machine-readable data included consensus sequences, predicted HIV drug resistance, and lists reporting the frequency of each mutation among sequenced reads.

### Phylogenetic analysis

Phylogenetic analysis was performed for paired PR/RT and IN sequences generated by ViroSeq and the AB kit. Multiple pairwise sequence alignment was performed using MAFFT (v7.471). Pairwise Tamura-Nei 93 genetic distance was determined using MEGA7 (v7.0.26). Maximum-likelihood phylogenetic trees were constructed using RAxML (v8.2.12). Phylogenetic tools were accessed using the Cyber Infrastructure for Phylogenetic Research (CIPRES) Science Gateway. BLAST (https://blast.ncbi.nlm.nih.gov/Blast.cgi) was used to identify background sequences for phylogenetic trees; a maximum of 10 background sequences were identified for each study sequence. Background and reference sequences were obtained from the Los Alamos National Laboratory HIV Sequence Database (www.hiv.lanl.gov). HIV subtypes were obtained using the Stanford HIV Drug Resistance Database (v9.5.0).

## RESULTS

Ninety-six plasma samples were available for analysis with the AB kit (median viral load: 22,402 copies/mL, range: 530–737,741); 9.4% of the samples had a viral load below 2,000 copies/mL and 24.0% of samples had a viral load below 5,000 copies/mL. Results for both PR/RT and IN were obtained using the AB kit for 95 of the 96 samples (1 sample failed RNA extraction; viral load: 204,360). One or more steps of the analysis were repeated for 16 (16.8%) of the 95 samples (median viral load: 24,705 copies/mL, range: 840–362,620). In nine cases, sequencing was repeated successfully using previously generated nested PCR products (two for PR/RT, five for IN, two for both regions). In the other seven cases, reverse transcription/PCR was repeated using previously extracted RNA (median viral load: 24,960 copies/mL, range: 2,890–362,620). All 95 samples had HIV subtype B. Sequences generated with ViroSeq and the AB kit had a mean genetic distance of 0.02% for PR/RT (range: 0.00%–0.90%) and 0.04% for IN (range: 0.00%–0.94%); these data include results from participants who reported prior ART and for ART-naïve participants. In all cases, the sequences for each plasma sample that were generated with ViroSeq and the AB kit paired together on phylogenetic trees with bootstrap support ≥97% for PR/RT and ≥88% for IN.

One-hundred-three major RAMs were detected with both ViroSeq and the AB kit (39 [37.9%] NRTI RAMs, 27 [26.2%] NNRTI RAMs, 18 [17.5%] PI RAMs, 19 [18.4%] INSTI RAMs; Table 1). Four additional major RAMs were detected by the AB kit only (two NNRTI RAMs: E138A and G190A; one NRTI RAM: D67N; and one INSTI RAM: E92Q). Two of these mutations, G190A and E92Q, independently predict high-level resistance to at least one ARV drug. In one case, G190A was detected in a sample that also had E138A detected by both assays; detection of G190A by the AB kit changed the resistance status from no resistance to high-level NNRTI resistance. In the other case, E92Q was detected in a sample that also had four other major RAMs detected by both assays; detection of E92Q changed the resistance status from high-level resistance to the INSTIs cabotegravir, elvitegravir, and raltegravir to high-level resistance to the INSTI dolutegravir in addition to the other three drugs.

**TABLE 1:** Major RAMs detected by ViroSeq and the AB kit^*a*^^,*b*^

NRTI	*N*	%	NNRTI	*N*	%	PI	*N*	%	INSTI	*N*	%
M184V	15	38.50	K103N	14	51.90	V32I	3	16.70	E92Q	6	31.60
K65R	4	10.30	E138A	2	7.40	M46I	2	11.10	S147G	4	21.10
M41L	4	10.30	E138K	2	7.40	M46L	2	11.10	E138K	3	15.80
L74I	2	5.10	G190A	2	7.40	I47V	2	11.10	T66A	1	5.30
M184I	2	5.10	L100I	1	3.70	I54L	2	11.10	G140S	1	5.30
Q151M	2	5.10	K101E	1	3.70	I84V	2	11.10	Y143C	1	5.30
T215Y	2	5.10	K103S	1	3.70	L90M	2	11.10	Q148H	1	5.30
Y115F	2	5.10	E138G	1	3.70	I54M	1	5.60	Q148R	1	5.30
D67N	1	2.60	Y181C	1	3.70	V82A	1	5.60	N155H	1	5.30
K70E	1	2.60	Y181I	1	3.70	V82T	1	5.60			
K70R	1	2.60	Y188L	1	3.70						
L210W	1	2.60									
K219E	1	2.60									
K219Q	1	2.60									

^*a*^ The table shows the 103 major RAMs detected by ViroSeq and the AB kit. The 103 RAMs included 39 NRTI RAMs, 27 NNRTI RAMs, 18 PI RAMs, and 19 INSTI RAMs.

^*b*^ Abbreviations: RAMs, resistance-associated mutations; ViroSeq, ViroSeq HIV-1 Genotyping System; AB kit, Applied Biosystems HIV-1 Genotyping Kit with Integrase; NRTI, nucleoside/nucleotide reverse transcriptase inhibitor; N, number; NNRTI, non-nucleoside reverse transcriptase inhibitor; PI, protease inhibitor; INSTI, integrase strand transfer inhibitor.

Seventy-two accessory RAMs were detected with both ViroSeq and the AB kit. Seven additional accessory RAMs were detected by the AB kit only (four NRTI mutations: D67G, K219R, T215I, T215I; one PI mutation: L10F; and two INSTI mutations: H51Y, G163R). Two additional accessory RAMs were detected by ViroSeq that were not detected by the AB kit (one NRTI mutation: E44D; one INSTI mutation: V151A). Detection of additional accessory RAMs by either kit did not impact the drugs with predicted high-level resistance.

NGS results were obtained for all 95 samples. An average of 188,808 NGS reads (range: 7,458–1,149,892) was generated in the PR/RT and IN regions for the samples tested. Paired NGS and ViroSeq consensus sequences had a mean genetic distance of 0.02% for PR/RT (range: 0.00%–0.93%) and 0.04% for IN (range: 0.00%–0.93%); NGS and AB kit consensus sequences had a mean genetic distance of 0.00% for PR/RT (range: 0.00%–0.00%) and 0.00% for IN (range: 0.00%–0.12%). Of the 103 major RAMs detected by both Sanger-based genotyping systems (ViroSeq and the AB kit), 102 were also detected by NGS; 1 NRTI mutation, M184I, was detected by ViroSeq and the AB kit but not by NGS. This discrepancy reflects the presence of multiple nucleic acid mixtures at this position. NGS detected two codons at position 184 in RT: ATG, which encodes methionine (detected in 73.1% of reads) and GTA, which encodes valine (detected in 25.4% of reads). The codon in the consensus sequences generated by ViroSeq and the AB kit was RTR (R = A + G); the Stanford HIV Drug Resistance Database interpreted this codon mixture as including ATA, which encodes isoleucine (e.g., M184I).

Table 2 shows the major RAMs that were detected by only one or two of the three methods (ViroSeq, the AB kit, and NGS). The four major RAMs that were detected by the AB kit but not by ViroSeq were also detected by NGS; the frequency of these mutations among sequenced reads ranged from 18.1% to 38.2%. Twenty-seven major RAMs were detected by NGS that were not detected by either of the Sanger-based systems (9 NRTI RAMs, 3 NNRTI RAMs, 5 PI RAMs, and 10 INSTI RAMs); the mutation frequency among sequenced reads ranged from 2.0% to 13.8%. Seventeen (63.0%) of the 27 major RAMs that were detected by NGS only independently predict high-level resistance to at least one ARV drug; the frequency of the 17 mutations among sequenced reads ranged from 2.2% to 9.7%. The 27 major RAMs detected by NGS only were detected in samples from 21 individuals. These RAMs impacted resistance status as follows: in six cases, resistance status changed from no resistance to resistant; in six cases, the additional RAMs predicted resistance to an additional drug class; in three cases, the additional RAMs increased resistance to one or more drugs in the same drug class; and in six cases, there was no change in resistance status.

**TABLE 2:** Major RAMs that were differentially identified using ViroSeq, the AB kit, and a laboratory-developed NGS method^*a*^^*,^b^*^

Drug class	Major RAM	ViroSeq	AB	NGS	NGS reads % (*N*/total)
NRTI	D67N	✗	✓	✓	18.10 (34,870/192,612)
	M41L	✗	✗	✓	8.63 (18,199/210,977)
	M184V	✗	✗	✓	7.13 (11,870/166,524)
	M184V	✗	✗	✓	4.76 (9,647/202,868)
	K70R	✗	✗	✓	4.19 (30,659/731,994)
	L210W	✗	✗	✓	2.96 (4,010/135,609)
	M184V	✗	✗	✓	2.75 (3,580/130,346)
	K70E	✗	✗	✓	2.62 (19,212/731,994)
	L210W	✗	✗	✓	2.46 (3,903/158,758)
	Y115F	✗	✗	✓	2.22 (3,182/143,089)
	M184I	✓	✓	✗	-
NNRTI	E138A	✗	✓	✓	38.18 (36,358/95,231)
	G190A	✗	✓	✓	21.69 (42,817/197,390)
	K101E	✗	✗	✓	13.83 (31,766/229,662)
	K103N	✗	✗	✓	9.73 (21,575/221,779)
	G190E	✗	✗	✓	2.92 (5,544/189,626)
PI	D30N	✗	✗	✓	3.78 (12,672/335,452)
	D30N	✗	✗	✓	3.20 (3,852/120,329)
	M46I	✗	✗	✓	2.50 (3,843/153,718)
	D30N	✗	✗	✓	2.33 (2,190/93,975)
	V82A	✗	✗	✓	2.04 (3,858/189,361)
INSTI	E92Q	✗	✓	✓	18.71 (81,492/435,526)
	Q148R	✗	✗	✓	6.55 (8,094/123,633)
	Y143S	✗	✗	✓	5.73 (12,529/218,764)
	Y143S	✗	✗	✓	4.84 (8,505/175,790)
	Y143S	✗	✗	✓	3.53 (4,302/122,033)
	Y143S	✗	✗	✓	3.19 (8,940/280,023)
	P145S	✗	✗	✓	3.15 (5,023/159,541)
	Q148K	✗	✗	✓	2.39 (5,792/242,724)
	R263K	✗	✗	✓	2.30 (5,143/223,470)
	E92Q	✗	✗	✓	2.24 (4,121/184,148)
	Q148K	✗	✗	✓	2.20 (4,791/217,886)

^*a*^ The table shows major RAMs that were not detected by at least one sequencing method (ViroSeq, AB kit, or NGS). The 32 major RAMs were detected among samples from 22 participants (4 participants had 3 mutations detected, 2 participants had 2 mutations detected, and 16 had 1 mutation detected). The frequency of detection among NGS reads, the number of reads containing the mutation, and the total number of reads are shown for each mutation.

^*b*^ Abbreviations: RAMs, resistance-associated mutations; ViroSeq, ViroSeq HIV-1 Genotyping System; AB kit, Applied Biosystems HIV-1 Genotyping Kit with Integrase; NGS, next-generation sequencing; AB, AB kit; N, number; NRTI, nucleoside/nucleotide reverse transcriptase inhibitor; NNRTI, non-nucleoside reverse transcriptase inhibitor; PI, protease inhibitor; INSTI, integrase strand transfer inhibitor.

Seventy (97.2%) of the 72 accessory RAMs that were detected by both Sanger-based systems were also detected by NGS. One NRTI mutation (T215A) and one INSTI mutation (L74M) were detected by ViroSeq and the AB kit but not by NGS. As previously described, these discrepancies reflect the presence of multiple nucleic acid mixtures at this position. NGS detected three codons at position 215 in RT: GTC, which encodes valine (detected in 52.0% of reads), ACC, which encodes threonine (detected in 35.6% of reads), and ATC, which encodes isoleucine (detected in 11.1% of reads). The codon in the consensus sequences generated by the AB kit and ViroSeq was RYC (R = A + G, Y = C + T); this codon mixture includes GCC, which encodes alanine (e.g., T215A), which was not detected by NGS. NGS also detected three codons at position 74 in IN: CTG, which encodes leucine (detected in 49.3% of reads), ATA, which encodes isoleucine (detected in 39.3% of reads), and CTA, which encodes leucine (detected in 9.4% of reads). The codon in the consensus sequences generated by the AB kit and ViroSeq was MTR (M = A + C, R = A + G); this codon mixture includes ATG, which encodes methionine (e.g., L74M), which was not detected by NGS.

Of the seven accessory RAMs that were detected by the AB kit but not ViroSeq, six were also detected by NGS; mutation frequencies among sequenced reads ranged from 6.4% to 19.4%. One PI mutation (L10F) was not detected by NGS; this discrepancy was not due to the presence of multiple nucleic acid mixtures at this codon. Neither of the two accessory RAMs that were detected by ViroSeq but not by the AB kit were detected by NGS.

## DISCUSSION

This study evaluated the performance of the AB kit using a sample set with a high prevalence of HIV drug resistance mutations. This sample set was previously analyzed using the ViroSeq HIV-1 Genotyping System, which was discontinued in 2021 (8). Genotyping results for HIV PR, RT, and IN were obtained for all samples analyzed with the AB kit. Some samples (16/95; 16.8%) required repeat analysis due to initial Sanger sequencing failure. The viral load range of these samples was 840–362,620 RNA copies/mL; this suggests that low viral load was not the cause of sequencing failure. We were not able to determine the reason for the initial sequencing failure for these samples. This study demonstrates that performance of the AB kit for the detection of HIV drug resistance mutations is comparable to performance of the discontinued ViroSeq system. The AB kit identified all major RAMs previously identified with ViroSeq and detected four additional major RAMs not identified by ViroSeq, including two mutations that independently predict high-level drug resistance. The mutations identified by the AB kit include RAMs that predict resistance to dolutegravir (File S2), which is the backbone of first- and second-line HIV treatment regimens recommended by the World Health Organization (10).

This study also demonstrates that DNA amplicons from the AB kit can be used for NGS-based analysis of HIV drug resistance. NGS of AB kit amplicons identified 27 additional major RAMs that were not detected by the AB kit or ViroSeq, including 17 mutations that independently predict high-level drug resistance. These findings are consistent with prior research that demonstrates more sensitive detection of low-abundance RAMs (LA-RAMs) using NGS compared to Sanger sequencing (11).

In three cases, both of the Sanger sequencing methods (ViroSeq and the AB kit) identified mutations that were not detected using NGS; one of these mutations was a major RAM, and two were accessory RAMs. These mutations were identified by the Stanford HIV Drug Resistance Database Program at codon positions in the consensus sequences that included two ambiguous nucleotides (i.e., codons that included more than one nucleotide mixture). This type of sequence interpretation error occurs because the program cannot determine which mixed bases are linked and must report all possible nucleotide combinations at these positions. This issue does not occur with NGS-based analysis, highlighting a potential advantage of NGS-based resistance testing compared to Sanger-based methods.

While it is clear that NGS-based HIV genotyping methods are more sensitive for detecting LA-RAMs, the clinical significance of these mutations remains unclear (12). In 2020, the World Health Organization HIVResNet Working Group reviewed 103 studies that assessed prevalence, detection, and/or clinical significance of LA-RAMs in ARV drug-naïve adults (13). The clinical impact of LA-RAMs on first-line ART regimens was described in 42 (40.8%) of the 103 studies; a significant association between pre-treatment LA-RAMs and ART failure was observed in 16 studies but was not observed in the remaining 26 studies. Furthermore, some persons with pre-treatment LA-RAMs did not experience treatment failure, and pre-treatment LA-RAMs were often not selected in those failing treatment. Evaluation of data from these and prior studies has been complicated by variation in study design, methods used for mutation detection, and other factors.

There are many challenges to implementing the use of NGS for HIV drug resistance testing (14). Quality assessment and standardization of NGS methodologies are urgently needed to facilitate data comparison between studies (11, 15). There is also a need to define the optimal threshold for LA-RAMs for clinical applications (11, 13, 14, 16, 17). Several studies have reported that LA-RAMs detected at levels of 2–9% were linked to virologic failure (15, 18–21). Of the 27 major RAMs detected by NGS only in our study, 6 (22.2%) were detected at >5%, including 2 (7.4%) that were detected at >9%. While the use of NGS-based methods for HIV genotyping is increasing for both research and clinical applications, different methods for genotyping may be advantageous in different settings. Sanger-based HIV genotyping systems, like the AB kit and TaqPath kit, may be preferred for some research applications and clinical settings. Evaluation of LA-RAMs using NGS is also important in ART-naïve populations because RAMs can be transmitted and may be selected with use of ARV drugs for PrEP, treatment of hepatitis virus (22), and recreational purposes (23, 24).

This study has several limitations. First, all samples in this study had HIV-1 subtype B; further studies are needed to assess performance of the AB kit using other HIV-1 subtypes. Second, comprehensive information on ART regimens and treatment outcomes was not collected in the HPTN 078 study. Therefore, associations between detected RAMs and ART failure could not be evaluated. Third, errors may be introduced during PCR amplification or NGS (14, 15). The AB kit uses nested PCR amplification to generate amplicons, which may increase the possibility of PCR-based errors when these amplicons are used for NGS. We used a mutation detection cutoff of 2% for NGS analysis; current literature suggests that an NGS cutoff of 1–2% decreases the likelihood of detecting PCR- and sequencing-based errors (16, 25, 26). Fourth, due to limited sample volumes, we were not able to perform repeat/duplicate testing to evaluate the reproducibility of detection of mutations by NGS. Other approaches, such as the use of unique molecular identifiers during reverse transcription, could be used to assess the frequency of PCR- and sequencing-based errors in NGS data (13, 17, 26, 27). Finally, parameters required for clinical assay validation were not evaluated in this study since the AB kit is not cleared by the U.S. FDA for clinical use.

## Data Availability

Sequence data from ViroSeq, the AB kit, and NGS are available on GenBank (see File S1).

## SUPPLEMENTAL MATERIAL

Supplemental File 1 jcm.00136-24-s0001.docx GenBank accession numbers.

Supplemental File 2 jcm.00136-24-s0002.docx Major integrase strand transfer inhibitor RAMs and predicted dolutegravir resistance.

ASM does not own the copyrights to Supplemental Material that may be linked to, or accessed through, an article. The authors have granted ASM a non-exclusive, world-wide license to publish the Supplemental Material files. Please contact the corresponding author directly for reuse.
