# Supplementary material for: Performance of the Applied Biosystems HIV-1 Genotyping Kit with Integrase
Source: J Clin Microbiol. 2024 May 10;62(6):e00136-24. doi: 10.1128/jcm.00136-24 (PMC11237527; doi:10.1128/jcm.00136-24)
Supplement: Supplemental File 1 — GenBank accession numbers. [file jcm.00136-24-s0001.docx]

**SUPPLEMENTAL FILE 1**

Table: GenBank accession numbers.

The table shows the accession numbers for consensus sequences available in the GenBank sequence database (https://www.ncbi.nlm.nih.gov/genbank/).

| **Genotyping method** | **Region** | **GenBank accession numbers** |
| --- | --- | --- |
| ViroSeq | PR/RT | MK580178, MK580179, MK580181, MK580182, MK580184, MK580186, MK580190-MK580192, MK580194, MK580195, MK580197, MK580198, MK580201, MK580202, MK580205, MK580208, MK580210, MK580215-MK580233, MK580235-MK580238, MK580240-MK580252, MK580254-MK580258, MK580261, MK580262, MK580264-MK580266, MK580269, MK580271-MK580288, MK580290-MK580298, MK580301, MK580305, MK580308 |
|  | IN | MK580320, MK580321, MK580323, MK580324, MK580326, MK580327, MK580331-MK580333, MK580335, MK580336, MK580338, MK580339, MK580342, MK580343, MK580346, MK580349, MK580351, MK580355-MK580373, MK580375-MK580378, MK580380-MK580392, MK580394-MK580398, MK580400-MK580404, MK580407, MK580409-MK580426, MK580428-MK580436, MK580439, MK580443, MK580446 |
| AB kit | PR/RT | PP084425 - PP084519 |
|  | IN | PP084520 - PP084614 |
| NGS | PR/RT | PP097457 - PP097551 |
|  | IN | PP097552 - PP097646 |

Abbreviations: ViroSeq: ViroSeq HIV-1 Genotyping System; AB kit: Applied Biosystems HIV-1 Genotyping Kit with Integrase; NGS: next-generation sequencing; PR/RT: protease/reverse transcriptase; IN: integrase.
