# Supplementary material for: Performance of the Applied Biosystems HIV-1 Genotyping Kit with Integrase
Source: J Clin Microbiol. 2024 May 10;62(6):e00136-24. doi: 10.1128/jcm.00136-24 (PMC11237527; doi:10.1128/jcm.00136-24)
Supplement: Supplemental File 2 — Major integrase strand transfer inhibitor RAMs and predicted dolutegravir resistance. [file jcm.00136-24-s0002.docx]

**SUPPLEMENTAL FILE 2**

Table: Major integrase strand transfer inhibitor resistance-associated mutations and predicted dolutegravir resistance.

The table shows the major integrase strand transfer inhibitor (INSTI) resistance-associated mutations (RAMs) detected by the Applied Biosystems HIV-1 Genotyping Kit with Integrase and/or the ViroSeq HIV-1 Genotyping System. The predicted resistance to dolutegravir (DTG) is shown for individual RAMs and collectively for participants with multiple INSTI RAMs detected. DTG resistance was predicted using the Stanford HIV Drug Resistance Database.

| **Participant** | **Mutation** | **AB kit** | **ViroSeq** | **Predicted DTG**  **resistance**  (by mutation) | **Predicted DTG resistance**  (by participant) |
| --- | --- | --- | --- | --- | --- |
| 1 | E92Q | yes | yes | potential low-level resistance | high-level resistance |
|  | E138K | yes | yes | potential low-level resistance |  |
|  | S147G | yes | yes | potential low-level resistance |  |
|  | N155H | yes | yes | potential low-level resistance |  |
| 2 | E92Q | yes | yes | potential low-level resistance | - |
| 3 | T66A | yes | yes | susceptible | high-level resistance |
|  | E92Q | yes | no | potential low-level resistance |  |
|  | E138K | yes | yes | potential low-level resistance |  |
|  | Y143C | yes | yes | susceptible |  |
|  | S147G | yes | yes | potential low-level resistance |  |
| 4 | E92Q | yes | yes | potential low-level resistance | low-level resistance |
|  | S147G | yes | yes | potential low-level resistance |  |
| 5 | E92Q | yes | yes | potential low-level resistance | - |
| 6 | E92Q | yes | yes | potential low-level resistance | - |
| 7 | E92Q | yes | yes | potential low-level resistance | - |
| 8 | E138K | yes | yes | potential low-level resistance | high-level resistance |
|  | S147G | yes | yes | potential low-level resistance |  |
|  | Q148R | yes | yes | low-level resistance |  |
| 9 | G140S | yes | yes | potential low-level resistance | intermediate resistance |
|  | Q148H | yes | yes | low-level resistance |  |

Abbreviations: AB kit: Applied Biosystems HIV-1 Genotyping Kit with Integrase; ViroSeq: ViroSeq HIV-1 Genotyping System; DTG: dolutegravir.
